# Forecast of a future leveling of the incidence trends of female breast cancer in Taiwan: an age-period-cohort analysis

**DOI:** 10.1038/s41598-022-16056-y

**Published:** 2022-07-21

**Authors:** Yi-Chu Chen, Shih-Yung Su, Jing-Rong Jhuang, Chun-Ju Chiang, Ya-Wen Yang, Chao-Chun Wu, Li-Ju Lin, Wen-Chung Lee

**Affiliations:** 1 Institute of Epidemiology and Preventive Medicine, College of Public Health, National Taiwan University, Rm. 536, No. 17, Xuzhou Rd., Taipei, 100 Taiwan; 2 Innovation and Policy Center for Population Health and Sustainable Environment, College of Public Health, National Taiwan University, Taipei, Taiwan; 3 Taiwan Cancer Registry, Taipei, Taiwan; 4 Health Promotion Administration, Taipei, Taiwan

**Keywords:** Diseases, Oncology, Mathematics and computing

## Abstract

Breast cancer is the most common cancer among women in Taiwan. The age-standardized incidence rate has doubled in just 20 years, causing considerable concern to health professionals and the general public. This study used an ensemble of age-period-cohort models to estimate breast cancer incidence trends in Taiwan from 1997 to 2016 and project trends up to 2035. The (truncated) world standard population (World Health Organization 2000) proportions (age groups: 25–29, 30–34, …, 80–84, and older than 85 years) were used to calculate age-standardized incidence rates. The age-standardized incidence rate from 1997 (60.33/100,000 population) to 2016 (128.20/100,000 population) increased rapidly. The projection is that the increase in the age-standardized incidence will subsequently slow and exhibit a plateau in 2031 (151.32/100,000 population). From 2026 to 2035, the age-specific incidence rates for women older than 55 years old (postmenopausal breast cancer) are projected to increase with larger percentage increments for older women. A future leveling of female breast cancer incidence trends in Taiwan is anticipated. The majority of the patients with breast cancer in the future will be women aged 55 years and older. Education on lifestyle recommendations and mammography screening is required to reduce the burden of breast cancer. The results should have implications for other countries which are also confronted with the same public health problem of rapidly increasing breast cancer incidences.

## Introduction

Would Health Organization announce that breast cancer has overtaken lung cancer as the most common cancer in the world^1^. In Taiwan, it is also the leading cancer among women. The age-standardized incidence rate of breast cancer in Taiwan has increased in recent decades, doubling in just 20 years from 1997 to 2016. Such rapid rises constitute a significant concern to health professionals and the general public. In response to this crisis, the Health Promotion Administration in Taiwan launched a nationwide mammography screening program (every two years for women aged 50 to 69) in 2004. In 2009, the program was further expanded to cover 45 to 69 year old women and 40 to 44 year old women with second-degree relatives ever diagnosed with breast cancer.

Secular trends may provide clues about the etiological factors for breast cancer, generating hypotheses that can be tested. Examination of the patterns and trends of breast cancer incidences enables the etiology of the illness and targets for intervention to be identified to mitigate the disease’s burden. Cancer incidence projections are also essential for the planning of resources and the informing of cancer control programs. In this paper, we fit an ensemble of age-period-cohort (APC) models to breast cancer incidence data in Taiwan from 1997 to 2016 and provide incidence rate projections up to 2035 to establish whether the increasing trend will continue increasing or will level off. The results should be implicative for other countries also confronted with the same public health problem of rapidly growing breast cancer incidences.

## Materials and methods

### Data source

Breast cancer cases (The International Statistical Classification of Diseases and Related Health Problems 10th Revision: C50) in women and the corresponding populations were abstracted from the Taiwan Cancer Registry. Some quality indicators for this registry are as follows: population coverage = 98.4%; percentage of cases with death certificate only = 0.9%; percentage of morphological verification = 93.0% for all sites combined; data timeliness = 14 months^2,3^, which rank the registry among the top-tier gold-level cancer registries in the world. Supplementary Table 1 lists the data quality indices of the Taiwan Cancer Registry from 2002 to 2016.

We used 13 groups to categorize the age at diagnosis of patients with breast cancer aged 25 years and older (25–29, 30–34, …, 80–84, and more than 85 years). Data from patients under 25 years at diagnosis were not used because of the scarcity of such cases (Supplementary Table 2). We categorized the calendar years of diagnosis according to each year from 1997 to 2016 by every year (20 groups). The (truncated) world standard population (World Health Organization 2000) proportions (age groups: 25–29, 30–34, …, 80–84, and older than 85 years; Supplementary Table 3) were used to calculate age-standardized incidence rates. The population projection method was the cohort-component method, and the source was National Development Council Population Projections for Taiwan.

This study protocol was approved by the National Taiwan University Research Ethics Committee (202101HM030) and the Data Release Review Board of the Health Promotion Administration, Ministry of Health and Welfare in Taiwan. All methods were performed in accordance with the relevant guidelines and regulations. In addition, the Research Ethics Committee waived the requirement for informed consent due to the lack of personal information and secondary data in the study.

### Statistical analysis

We used the APC model to analyze the breast cancer incidence rate and project the trend to 2035. Because of the perfect collinearity between the three temporal factors (cohort = period – age), the APC model suffers from a non-identification problem. In addition, we used data provided in 5-year age groups and 1-year periods in the study, which might cause additional identifiability issues with the unequal intervals in the definition of cohort indices^4^. However, we considered the APC model with the cohort curvature effect but without the linear cohort effect for making parameters estimable. The incidence projections in this study were impervious to the non-identifiability problem because the fitted values in the nonidentifiable APC model were the same for all possible sets of parameter estimates.

Details of the methods we used in this study were described elsewhere^5^. An ensemble of 265 APC models was first constructed using the breast cancer incidence data from 1997 to 2006. The 53 model types, consisting of cubic spline APC models (knots were placed at percentiles)^6^, polynomial APC models (quadratic, cubic, and other polynomial models)^7^, and Tzeng and Lee’s APC model^8^, were presented in Supplementary Table 4. The constructed APC models were then used to predict breast cancer incidence rates from 2007 to 2016 with 5 types of link functions (log, power 2, power 3, power 4, and power 5). The prediction was further subjected to a 0%, 5%, 10%, 15%, …, or 100% year-on-year attenuation, respectively, resulting in 5565 (53 model types × 5 link functions × 21 attenuations) sets of projection results.

The symmetric mean absolute percentage error (SMAPE) index was used to quantify the prediction error for each projection. The model and the attenuation factor with the smallest SMAPE were selected. The selected model was then fitted to all available breast cancer incidence data from 1997 to 2016. Based on this refitted model and the previously selected attenuation factor, a projection was made for 2035; the age-specific incidence rates were first projected, and the age-standardized incidence rate was then calculated. For this model selection procedure, the model with the smallest cross-validation error was assumed to be appropriate for future projection^9,10^. Data management and analyses were performed using SAS statistical software (version 9.4).

## Results

The age-standardized breast cancer incidence rate increased from 60.35 to 128.20 per 100,000 population from 1997 to 2016 (the annual percentage change = 5.62%). The mean age of breast cancer diagnosis in Taiwan also increased from 50.5 years in 1997 to 56.0 years in 2016, and the median age at diagnosis was from 48 to 55 years old (Supplementary Table 5).

The best-selected model for the projection (SMAPE = 5.58%) was a polynomial APC model with a log link function and 30% attenuation. Table 1 presents the age-standardized (age range 25 + years) and age-specific incidence rates and case numbers of breast cancer with projected percentage changes from 2016 to 2025 and from 2026 to 2035. Supplementary Table 6 displays the projections of the age-standardized breast cancer incidence rates with different attenuations.

**Table 1:** Age-standardized and age-specific breast cancer incidence rates per 100,000 population (the observed rates in 2016 and the projected rates in 2025 and 2035) and the projected percentage changes from 2016 to 2025 and 2026 to 2035.

	Incidence rate in 2016	Case number in 2016	Projected incidence rate in 2025 (projected percentage change from 2016 to 2025)	Projected case number in 2025	Projected incidence rate in 2035 (projected percentage change from 2026 to 2035)	Projected case number in 2035
Age-standardized^a^	128.20		148.25 (15.64%)		149.45 (0.81%)	
**Age-specific**						
Age 25–29	9.48	73	9.99 (5.36%)	72.1	8.52 (− 14.71%)	40.1
Age 30–34	30.62	282	31.55 (3.02%)	247.3	27.64 (− 12.39%)	156.8
Age 35–39	70.24	719	76.11 (8.35%)	595.7	68.53 (− 9.96%)	505.5
Age 40–44	138.52	1270	150.36 (8.55%)	1434.6	139.33 (− 7.34%)	1100.8
Age 45–49	215.98	1992	217.55 (0.73%)	2218.8	207.68 (− 4.54%)	1621.6
Age 50–54	206.75	1956	244.90 (18.45%)	2187.8	241.13 (− 1.54%)	2276.6
Age 55–59	205.70	1832	251.68 (22.36%)	2287.9	255.58 (1.55%)	2564.2
Age 60–64	218.58	1732	259.51 (18.73%)	2383.0	272.66 (5.07%)	2378.0
Age 65–69	212.47	1163	259.81 (22.28%)	2197.6	282.40 (8.69%)	2472.5
Age 70–74	163.63	587	236.01 (44.23%)	1704.6	265.64 (12.55%)	2281.1
Age 75–79	168.47	536	205.05 (21.71%)	904.1	239.19 (16.65%)	1783.5
Age 80–84	140.55	305	199.31 (41.81%)	547.3	241.15 (20.99%)	1379.5
Age 85 +	111.23	205	160.06 (43.91%)	480.7	201.03 (25.60%)	920.2

^a^The World Health Organization’s 2000 world standard populations were used to compute the truncated age-standardized incidence rate (age range 25 + years).

An open interval of 85 + years was used in this paper instead of an equal 5-year age interval. For a sensitivity analysis, we restricted our analysis to the age range 25–84 years and projected the age-standardized and age-specific incidence rates and case numbers in Supplementary Table 7. Because there were very few cases aged over 85 + years, excluding them in the analysis is similar to when including them.

The age-standardized incidence rate of breast cancer was 128.20/100,000 population in 2016. The projected incidence rates are 148.25/100,000 population in 2025 (increased by 15.64% from 2016 to 2025) and 149.45/100,000 population in 2035 (increased by a mere 0.81% from 2026 to 2035). Age-specific incidence rates for all age groups are projected to increase from 2016 to 2025. The three age groups with the greatest increment are 70–74 years (increased by 44.23%), older than 85 years (increased by 43.91%), and 80–84 years (increased by 41.81%). By contrast, the age-specific incidence rate of the 45–49 year age group is projected to increase from 2016 to 2025 by only 0.73%. From 2026 to 2035, the age-specific incidence rate for women older than 55 is projected to increase further with larger percentage increments for older women.

**Figure 1:**
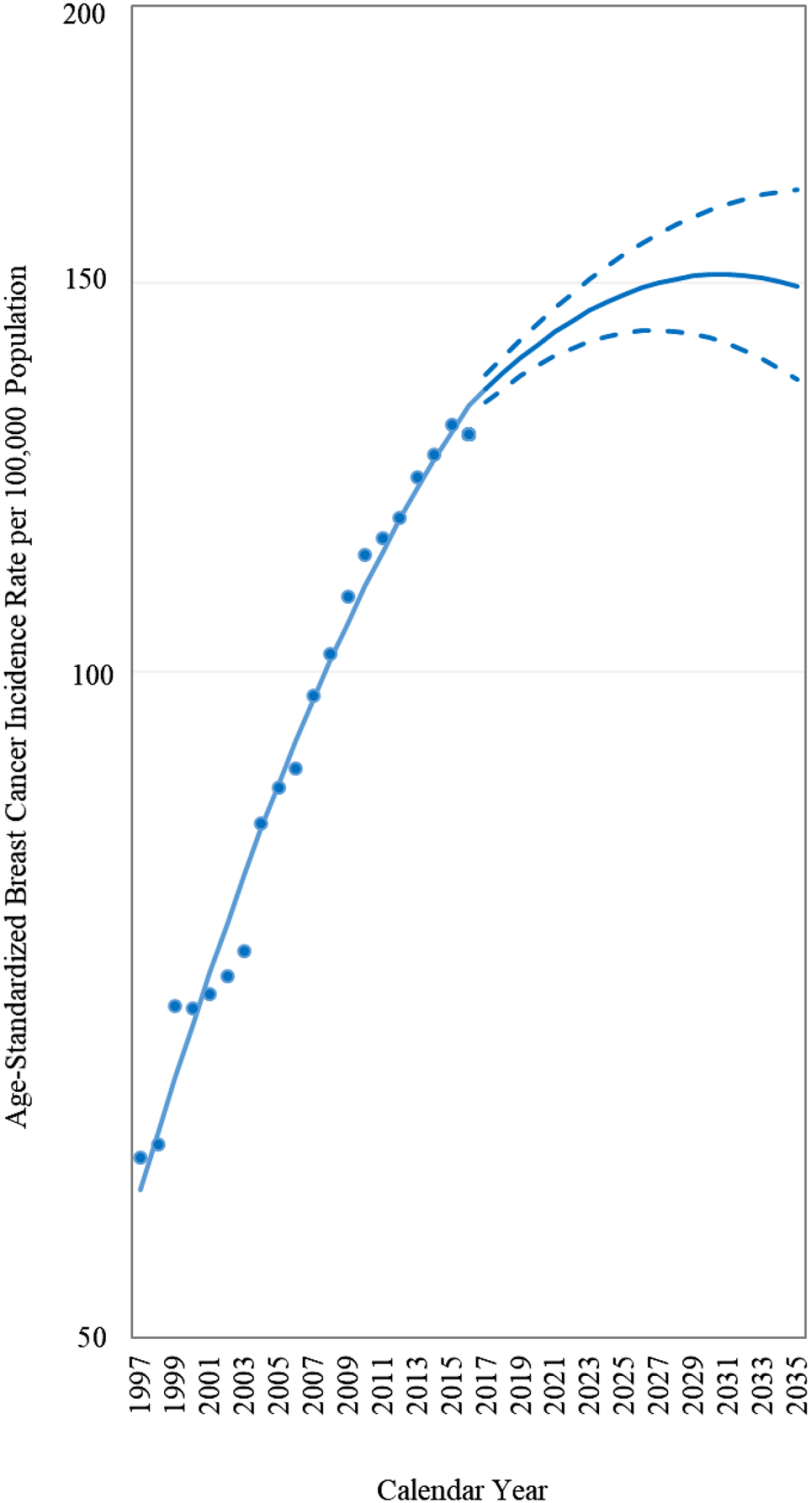
Age-standardized breast cancer incidence rate from 1997 to 2016 and projection from 2017 to 2035. The World Health Organization’s 2000 World Standard Populations were used to compute the truncated age-standardized incidence rate (age range 25 + years). Dotted lines indicate 95% confidence intervals for the projections.

Figure 1 displays the age-standardized breast cancer incidence rate from 1997 to 2016 and the projection from 2017 to 2035. The age-standardized incidence rate from 1997 (60.33/100,000 population) to 2016 (128.20/100,000 population) increased rapidly. The increase in the age-standardized incidence subsequently slows and exhibits a plateau in 2031 (151.32/100,000 population).

**Figure 2:**
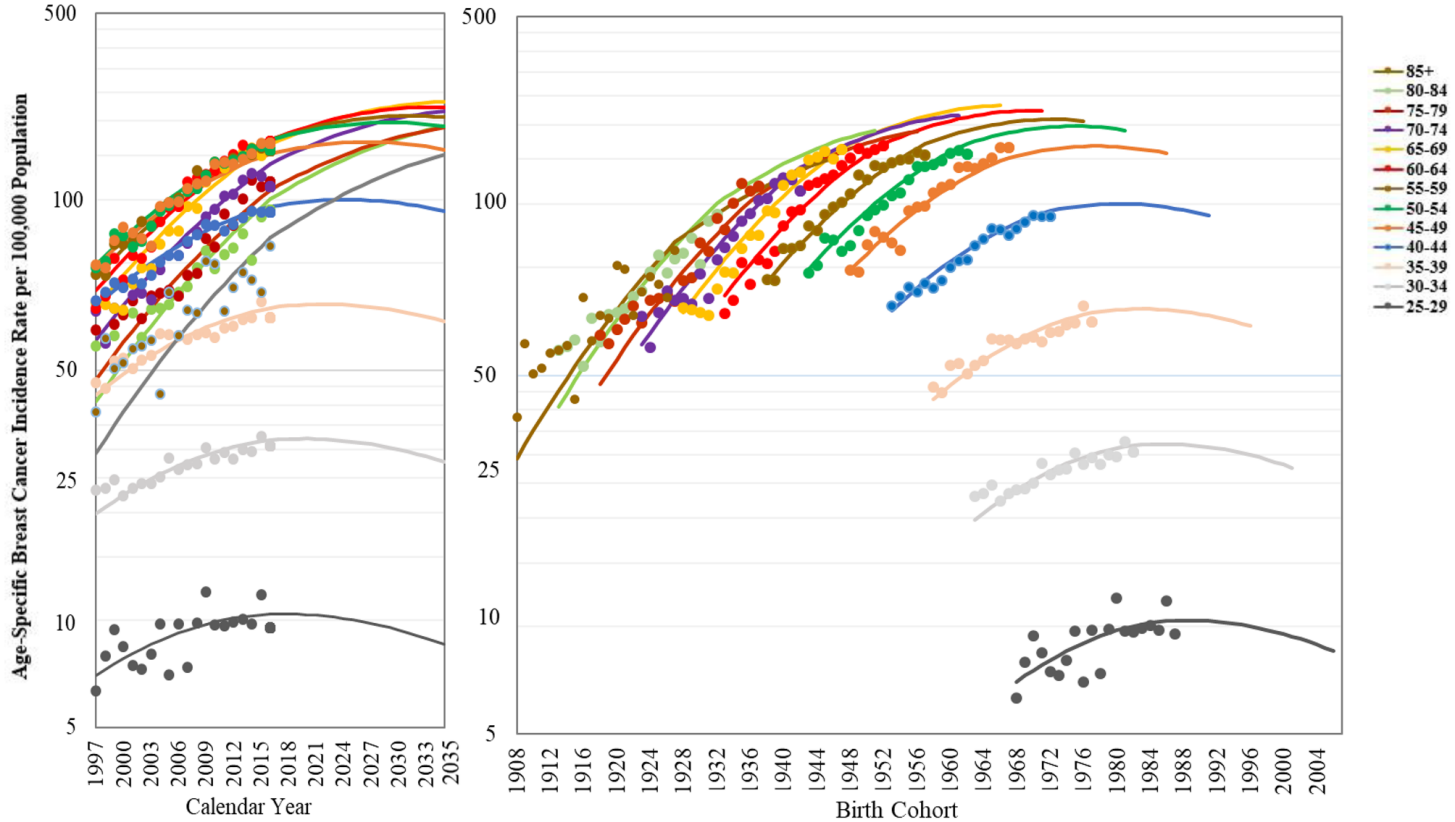
Age-specific breast cancer incidence rate from 1997 to 2016 and projections from 2017 to 2035 for calendar year and birth cohort.

Figure 2 demonstrates the age-specific breast cancer incidence rate from 1997 to 2016 and the projections from 2017 to 2035 for each calendar year and birth cohort. From 1997 to 2016, the age-specific incidence rate for each calendar year in all age groups has increased rapidly, particularly in women aged 60–64, 65–69, and older than 85 years. The projected age-specific incidence rates for each calendar year increase until 2035 for women older than 65 years. The projected age-specific incidence rates for each calendar year in the groups of women aged 50–54, 55–59, and 60–64 years exhibit a plateau in 2031 (247.46/100,000 population), 2031 (258.51/100,000 population), and 2033 (273.08/100,000 population), respectively. For women younger than 50 years, the projected age-specific incidence rates for each calendar year reach a plateau around 2017 to 2027. By comparison, the birth-cohort trends are more consistent across different ages. The age-specific incidence rates of the birth cohort born before 1985 increase rapidly, whereas the projected age-specific incidence rates of those born after 1985 reach a plateau.

## Discussion

This study observed a rapid increase in breast cancer incidence from 1997 to 2016. Taiwan has been industrialized since the 1960s. After 1960, Taiwanese women adapted to westernized lifestyles, resulting in higher caloric and higher fat diets, earlier menarche^11^, drastically reduced fertility rates^12^, later natural menopause ages^13^, and later childbearing ages. These are all documented risk factors for breast cancer^14,15^. Breast cancer incidence rapidly increased from 1997 to 2016, but the projected rate is more stable. Our finding shows that the projected age-standardized incidence rate of breast cancer in Taiwan will level off and exhibit a plateau in 2031 (151.32/100,000 population). Supplementary Figure S1 shows that (1) the age-standardized incidence rate for Taiwan in 1997 (60.33/100,000 population) was similar to that for Asian countries (China and India), (2) the rate for Taiwan in 2012 (117.29/100,000 population) was similar to that for Asian-Americans, and (3) the projected plateau rate for Taiwan in 2031 (151.32/100,000 population) is similar to that for Western developed countries or regions. (Supplementary Figure S2 also displayed the global age-standardized breast cancer incidence rates in 1997.) These indicate an epidemiological transition of breast cancer in Taiwan from Eastern countries to one characteristic of Western developed countries. We also projected the age-standardized breast cancer incidence rates separately for urban and rural regions in Taiwan into the future and found the trends leveling off in both regions (Supplementary Figure S3). We projected the age-standardized breast cancer mortality rate in Taiwan into the future (Supplementary Figure S4). However, Taiwan’s breast cancer mortality rate increases less dramatically than the incidence rate.

Age-specific incidence rates for all the age groups are projected to increase from 2016 to 2025. From 2026 to 2035, the age-specific incidence rate is projected to increase further for women older than 55. By contrast, the age-specific incidence rate for women younger than 55 is projected to plateau. In the future, most patients with breast cancer will be women aged 55 years and older (postmenopausal breast cancer). Differential trends among pre and postmenopausal women have been observed in many European countries, where the increases in incidence were more profound in women aged 50 to 69 years^16,17^. Asian women with breast cancer typically exhibit a younger onset age. The age curve of breast cancer incidence rates reaches a peak before 50 years^18^. This unimodal bell‐shaped age curve contrasts with the monotonic increase in age-specific breast cancer incidence rate observed in Western countries or regions^19^. Physicians and the general public in Taiwan believe that patients are diagnosed with breast cancer at increasingly young ages. The impression, however, is not valid. The mean age of breast cancer diagnosis in Taiwan increased from 51 years in 1997 to 56 years old in 2016 (and the median age at diagnosis from 48 to 55 years old). Shifts toward older peak ages of incidence have also occurred in Thailand^20^ and China^21^. Another explanation for the rise in premenopausal breast cancer incidence is screening policies^17^. Most North American and European countries recommend mammography examinations for women older than 50. In Taiwan, however, the recommended age to initiate mammography screening is an earlier age of 40 years old.

Breast cancer encompasses several distinct pathologic entities broadly divided into premenopausal and postmenopausal diseases^22^. Obesity increases breast cancer risk among postmenopausal women^23–25^. Associations between obesity and postmenopausal breast cancer are mediated mainly by serum estrogen levels^25^. Obesity is an established risk factor for postmenopausal breast cancer, not premenopausal breast cancer. This could explain some of the differences in age-specific incidence trends. The previous population-based survey from 1993 to 2008 indicated that obesity prevalence has increased among Taiwanese women^26^. Our projection study, which was based on the assumption that the breast cancer risk factors remain unchanged, suggests that the burden of the disease will continue to increase. This highlights questions concerning future health care infrastructure needs and possible preventive strategies.

Nevertheless, most breast cancer risk factors, including age at first birth, breastfeeding duration, obesity, alcohol use, oral contraceptive use, and hormone replacement therapy, are modifiable. While these are all potentially modifiable, it is unlikely that female reproductive cultures will change to prevent breast cancer, especially when this is balanced with women spending more years in education and entering the job market. Obesity is a more manageable modifiable risk factor. Studies have also indicated that obesity is the most dominant attributable risk factor among Iranian and Korean women^27,28^. Lifestyle changes also reportedly prevent 25%-30% of breast cancer cases^29^. Education campaigns regarding lifestyle recommendations are essential for reducing the burden of breast cancer.

Mammography screening may increase in situ breast cancer incidence rate and reduce invasive breast cancer incidence through early detection of breast tumors^30–32^. Mammography screening became widely used during the 1980s, and the coverage rate was estimated to be more than 70% in the United States and Western developed countries or regions^33^. However, Taiwan had no nationwide mammography screening programs until 2004^34^. The coverage rate was relatively low (only 40.9% of women aged 45–69 years received mammography in 2018)^35^ compared with the United States and Western developed countries or regions^33^. The percentage of patients with earlier stages of breast cancer only increased slightly in Taiwan (Supplementary Figure S5). Education campaigns on mammography screening are required to reduce the burden of invasive breast cancer.

A previous study pointed out a substantial birth-cohort effect in breast cancer incidence trend among Taiwanese women^36^. Similarly, we found a rapid increase from 1997 to 2016 (the birth-cohort born before 1985) and projected that the incidence would reach a plateau in the future (the birth-cohort born after 1985). The APC model is more suitable than other period-based projection models, such as joinpoint regression^37^, for projecting breast cancer incidence rates in Taiwan. (Supplementary Figure S6 compares the projections of breast cancer age-standardized incidence rate by the APC model and the joinpoint regression model.) Women born after 1985 (with the projected incidence rate reaching a plateau), while they were still young nowadays, will dominate the age-standardized incidence rate of breast cancer in the future (and lead the age-standardized rate to the plateau). The APC model we used in this study takes this into account and can provide advance notice of the age-standardized incidence trend leveling off. Many Asian countries, such as South Korea, Japan, Thailand, India, and China, suffer similar rapid increases in breast cancer incidence due to cohort effects^17,38^. We recommend these Asian countries (and any other country with an increased breast cancer incidence trend) use our method to make future projections of the incidence rate of breast cancer to see if the trend will continue increasing or level off.

A strength of our study is the use of high-quality population-based data to provide a comprehensive projection of breast cancer incidence rates. However, our study also has limitations. The lack of breast cancer molecular subtypes (estrogen receptor, progesterone receptor, and human epidermal growth factor receptor 2 status) and cancer stages prevented performing a more refined analysis. The study is ecological, and the inference is susceptible to the ecological fallacy^39^. Our model assumed that the distribution of risk factors (westernized lifestyles, obesity, reduced fertility, etc.) and public health policy (regarding breast cancer control) do not change over time. We did not consider the scenarios, for example, that the mammography coverage is raised to a level in the future comparable to those of most western countries nowadays. Finally, our APC projection model assumes no interaction between the three temporal factors. For example, if one birth cohort has twice the breast cancer risk as the reference birth cohort, each age group’s risk is twice that of the corresponding age group in the reference birth cohort. The assumption may fail, however.

In conclusion, a rapid increase in breast cancer incidence from 1997 to 2016 is noted. However, the projected age-standardized incidence rate of breast cancer in Taiwan will level off and exhibit a plateau in 2031. In the future, most breast cancer patients will be women aged 55 years and older (postmenopausal breast cancer). Education on lifestyle recommendations and mammography screening is required to reduce the burden of breast cancer incidence.

## Supplementary Information

Supplementary Information.

## Data Availability

This study uses restricted secondary data and, therefore, we cannot provide the dataset required to recreate our study.
