# Supplementary material for: Forecast of a future leveling of the incidence trends of female breast cancer in Taiwan: an age-period-cohort analysis
Source: Sci Rep. 2022 Jul 21;12:12481. doi: 10.1038/s41598-022-16056-y (PMC9304355; doi:10.1038/s41598-022-16056-y)
Supplement: Supplementary file 1 — Supplementary Information. [file 41598_2022_16056_MOESM1_ESM.pdf]

Title: Forecast of a Future Leveling of the Incidence Trends of Female Breast Cancer in Taiwan: An Age-Period-Cohort Analysis.

Authors: Yi-Chu Chen[1], Shih-Yung Su[1,2], Jing-Rong Jhuang[1,3], Chun-Ju Chiang[1,3], Ya-Wen Yang[3], Chao-Chun Wu[4], Li-Ju Lin[4], Wen-Chung Lee[1,2,3*]

Authors' affiliations:

[1] Institute of Epidemiology and Preventive Medicine, College of Public Health, National Taiwan University, Taipei, Taiwan

[2] Innovation and Policy Center for Population Health and Sustainable Environment, College of Public Health, National Taiwan University, Taipei, Taiwan

[3] Taiwan Cancer Registry, Taipei, Taiwan

[4] Health Promotion Administration, Taipei, Taiwan

*__Corresponding author__:

Professor Wen-Chung Lee, Institute of Epidemiology and Preventive Medicine, College of Public Health, National Taiwan University, Rm. 536, No. 17, Xuzhou Rd., Taipei 100, Taiwan. E-mail: wenchung@ntu.edu.tw

Supplement Table 1. Data quality indices for Taiwan cancer registry.

	2002	2003	2004	2005	2006	2007	2008	2009	2010	2011	2012	2013	2014	2015	2016
Completeness, %	92.8	95.5	94.3	96.7	97.6	97.8	97.6	97.6	97.0	97.6	98.4	98.4	98.2	98.2	98.4
DCO %[#]	2.9	2.6	2.3	1.7	1.4	1.3	1.2	1.1	0.9	0.8	0.7	0.9	0.9	92.6	93.0
M/I %[&]	54.8	54.4	55.6	51.8	50.2	50.7	47.4	44.7	44.4	45.9	44.5	44.4	44.7	43.8	45.2
MV %[$]	87.4	87.1	88.0	88.1	88.8	89.5	90.1	90.5	91.0	91.3	91.4	91.8	92.5	0.8	0.9
Timeliness, months	24	24	23	17	17	17	17	17	17	17	17	17	16	16	14

[#]DCO%: death certificate only (DCO) percentage;

[&]M/I: mortality to incidence ratio (only included invasive cancer cases)

[$]MV%: microscopically verified percentage

Supplement Table 2. The number of breast cancer patients aged less than 25 years old by the year of diagnosis in Taiwan.

Year of diagnosis	Case number
1997	17
1998	16
1999	22
2000	20
2001	20
2002	17
2003	22
2004	24
2005	16
2006	16
2007	16
2008	14
2009	15
2010	14
2011	11
2012	13
2013	15
2014	13
2015	16
2016	20

Supplement Table 3. The World Health Organization 2000 World Standard Population with the truncated age.

**Age Interval**	**Standard Population**	**Truncated Population**	**Truncated Population**
0-4	8,800	0	0
5-9	8,700	0	0
10-14	8,600	0	0
15-19	8,500	0	0
20-24	8,200	0	0
25-29	7,900	13,876	13,958
30-34	7,600	13,316	13,428
35-39	7,200	12,511	12,721
40-44	6,600	11,531	11,661
45-49	6,000	10,569	10,600
50-54	5,400	9,396	9,541
55-59	4,600	7,961	8,127
60-64	3,700	6,509	6,537
65-69	3,000	5,179	5,300
70-74	2,200	3,867	3,887
75-79	1,500	2,660	2,650
80-84	900	1,592	1,590
85+	600	1,033	0
Total	100,000	100,000	100,000

Supplement Table 4. The 53 types of age-period-cohort models in the ensemble.

Type	Age	Period	Cohort	Type	Age	Period	Cohort	Type	Age	Period	Cohort
poly1	a, a^2	p, p^2	c^2	res1	2 knots	2 knots	2 knots	res24	4 knots	3 knots	4 knots
poly2	a, a^2	p	c^2	res2	3 knots	2 knots	2 knots	res25	2 knots	3 knots	5 knots
poly3	a, a^2, a^3	p, p^2, p^3	c^2, c^3	res3	4 knots	2 knots	2 knots	res26	3 knots	3 knots	5 knots
poly4	a, a^2, a^3	p, p^2, p^3	c^2	res4	2 knots	2 knots	3 knots	res27	4 knots	3 knots	5 knots
poly5	a, a^2, a^3	p, p^2	c^2, c^3	res5	3 knots	2 knots	3 knots	res28	2 knots	3 knots	6 knots
poly6	a, a^2, a^3	p, p^2	c^2	res6	4 knots	2 knots	3 knots	res29	3 knots	3 knots	6 knots
poly7	a, a^2, a^3	p	c^2	res7	2 knots	2 knots	4 knots	res30	4 knots	3 knots	6 knots
poly8	a, …, a^4	p, p^2, p^3	c^2, c^3	res8	3 knots	2 knots	4 knots				
poly9	a, …, a^4	p, p^2, p^3	c^2	res9	4 knots	2 knots	4 knots				
poly10	a, …, a^4	p, p^2	c^2, c^3	res10	2 knots	2 knots	5 knots				
poly11	a, …, a^4	p, p^2	c^2	res11	3 knots	2 knots	5 knots				
poly12	a, …, a^4	p	c^2	res12	4 knots	2 knots	5 knots				
poly13	a, …, a^5	p, p^2, p^3	c^2, c^3	res13	2 knots	2 knots	6 knots				
poly14	a, …, a^5	p, p^2, p^3	c^2	res14	3 knots	2 knots	6 knots				
poly15	a, …, a^5	p, p^2	c^2, c^3	res15	4 knots	2 knots	6 knots				
poly16	a, …, a^5	p, p^2	c^2	res16	2 knots	3 knots	2 knots				
poly17	a, …, a^5	p	c^2	res17	3 knots	3 knots	2 knots				
poly18	orthogonal a	p, p^2, p^3	c^2, c^3	res18	4 knots	3 knots	2 knots				
poly19	orthogonal a	p, p^2, p^3	c^2	res19	2 knots	3 knots	3 knots				
poly20	orthogonal a	p, p^2	c^2, c^3	res20	3 knots	3 knots	3 knots				
poly21	orthogonal a	p, p^2	c^2	res21	4 knots	3 knots	3 knots				
poly22	orthogonal a	p	c^2	res22	2 knots	3 knots	4 knots				
Tseng	Linear + Curvature	Linear + Curvature	Curvature	res23	3 knots	3 knots	4 knots				

Supplement Table 5. The age-standardized breast cancer incidence rates per 100,000 population, mean and median ages of a breast cancer diagnosis from 1997 to 2016 in Taiwan.

	Age-standardized incidence rate#	Mean age of breast cancer diagnosis	Median age of breast cancer diagnosis
1997	60.35	50.5	48
1998	61.18	50.5	48
1999	70.71	50.6	48
2000	70.48	50.8	49
2001	71.56	51.5	49
2002	72.86	51.6	50
2003	74.81	51.9	50
2004	85.54	51.9	50
2005	88.74	52.3	51
2006	90.46	52.5	51
2007	97.63	53.1	52
2008	101.95	53.3	52
2009	108.23	53.8	52
2010	112.96	54.1	53
2011	114.98	54.5	53
2012	117.47	54.8	54
2013	122.55	55.2	54
2014	125.43	55.4	54
2015	129.47	55.4	54
2016	128.20	56.0	55

#The World Health Organization's 2000 world standard populations were used to compute the truncated age-standardized incidence rate (age range 25+ years)

Supplement Table 6. Projections of the age-standardized# breast cancer incidence rates per 100,000 population with different attenuations.

	0%	5%	10%	15%	20%	25%	30%	35%	40%	45%	50%	55%	60%	65%	70%	75%	80%	85%	90%	95%	100%	105%	110%
2017	134.10	133.93	133.77	133.61	133.44	133.28	133.11	132.95	132.78	132.62	132.46	132.29	132.13	131.96	131.80	131.63	131.47	131.30	131.14	130.98	130.81	130.65	130.48
2018	138.57	138.24	137.92	137.59	137.26	136.93	136.61	136.28	135.95	135.63	135.30	134.97	134.64	134.32	133.99	133.66	133.33	133.01	132.68	132.35	132.02	131.70	131.37
2019	141.59	141.11	140.63	140.15	139.67	139.20	138.72	138.24	137.76	137.28	136.81	136.33	135.85	135.37	134.89	134.41	133.94	133.46	132.98	132.50	132.02	131.55	131.07
2020	144.41	143.79	143.17	142.55	141.93	141.31	140.69	140.07	139.46	138.84	138.22	137.60	136.98	136.36	135.74	135.12	134.50	133.88	133.26	132.64	132.02	131.40	130.79
2021	147.03	146.28	145.53	144.78	144.03	143.28	142.53	141.78	141.03	140.28	139.53	138.78	138.03	137.28	136.53	135.77	135.02	134.27	133.52	132.77	132.02	131.27	130.52
2022	149.43	148.56	147.69	146.82	145.95	145.08	144.21	143.34	142.47	141.60	140.73	139.86	138.98	138.11	137.24	136.37	135.50	134.63	133.76	132.89	132.02	131.15	130.28
2023	151.59	150.62	149.64	148.66	147.68	146.70	145.72	144.74	143.77	142.79	141.81	140.83	139.85	138.87	137.90	136.92	135.94	134.96	133.98	133.00	132.02	131.05	130.07
2024	153.52	152.44	151.37	150.30	149.22	148.15	147.07	146.00	144.92	143.85	142.77	141.70	140.62	139.55	138.47	137.40	136.32	135.25	134.17	133.10	132.02	130.95	129.87
2025	155.19	154.03	152.88	151.72	150.56	149.40	148.25	147.08	145.93	144.77	143.61	142.45	141.29	140.13	138.97	137.82	136.66	135.50	134.34	133.18	132.02	130.87	129.71
2026	156.61	155.38	154.15	152.92	151.69	150.46	149.23	148.00	146.77	145.54	144.32	143.09	141.86	140.63	139.40	138.17	136.94	135.71	134.48	133.25	132.02	130.80	129.57
2027	157.75	156.47	155.18	153.89	152.61	151.32	150.03	148.75	147.46	146.17	144.89	143.60	142.32	141.03	139.74	138.46	137.17	135.88	134.60	133.31	132.02	130.74	129.45
2028	158.62	157.29	155.96	154.63	153.30	151.97	150.64	149.31	147.98	146.65	145.32	143.99	142.66	141.33	140.00	138.67	137.34	136.01	134.68	133.35	132.02	130.69	129.36
2029	159.22	157.86	156.50	155.14	153.78	152.42	151.06	149.70	148.34	146.98	145.62	144.26	142.90	141.54	140.18	138.82	137.46	136.10	134.74	133.38	132.02	130.66	129.31
2030	159.53	158.15	156.78	155.40	154.03	152.65	151.28	149.90	148.53	147.15	145.78	144.40	143.03	141.65	140.27	138.90	137.52	136.15	134.77	133.40	132.02	130.65	129.27
2031	159.55	158.18	156.80	155.42	154.05	152.67	151.32	149.92	148.54	147.17	145.79	144.41	143.04	141.66	140.28	138.91	137.53	136.15	134.78	133.40	132.02	130.65	129.27
2032	159.30	157.93	156.57	155.21	153.84	152.48	151.12	149.75	148.39	147.03	145.66	144.30	142.93	141.57	140.21	138.84	137.48	136.12	134.75	133.39	132.02	130.66	129.30
2033	158.76	157.42	156.09	154.75	153.41	152.08	150.74	149.40	148.07	146.73	145.39	144.06	142.72	141.38	140.05	138.71	137.37	136.03	134.70	133.36	132.02	130.69	129.35
2034	157.94	156.65	155.35	154.06	152.76	151.46	150.17	148.87	147.58	146.28	144.98	143.69	142.39	141.10	139.80	138.50	137.21	135.91	134.62	133.32	132.02	130.73	129.43
2035	156.85	155.61	154.37	153.13	151.89	150.65	149.45	148.16	146.92	145.68	144.44	143.20	141.96	140.71	139.47	138.23	136.99	135.75	134.51	133.27	132.02	130.78	129.54

#The World Health Organization's 2000 world standard populations were used to compute the truncated age-standardized incidence rate (age range 25+ years)

Supplement Table 7. Age-standardized (age range 25-84 years) and age-specific breast cancer incidence rates per 100,000 population (observed in 2016 and projected in 2025 and 2035).

	Incidence rate in 2016	Case number in 2016	Projected incidence rate in 2025	Projected case number in 2025	Projected incidence rate in 2035	Projected case number in 2035
Age- standardized[#]	128.47		148.61		149.49	
Age-specific						
Age 25-29	9.48	73	9.67	70	8.159	38
Age 30-34	30.62	282	31.476	247	27.305	155
Age 35-39	70.24	719	73.856	578	65.963	487
Age 40-44	138.52	1270	150.044	1432	138.163	1092
Age 45-49	215.98	1992	217.931	2223	207.174	1618
Age 50-54	206.75	1956	242.587	2167	238.389	2251
Age 55-59	205.70	1832	252.682	2297	256.999	2578
Age 60-64	218.58	1732	266.353	2446	280.716	2448
Age 65-69	212.47	1163	262.341	2219	286.821	2511
Age 70-74	163.63	587	233.083	1684	264.637	2272
Age 75-79	168.47	536	220.551	972	260.301	1941
Age 80-84	140.55	305	197.49	542	242.52	1387

[#]The World Health Organization's 2000 world standard populations were used to compute the truncated age-standardized incidence rate (age range 25-84 years)

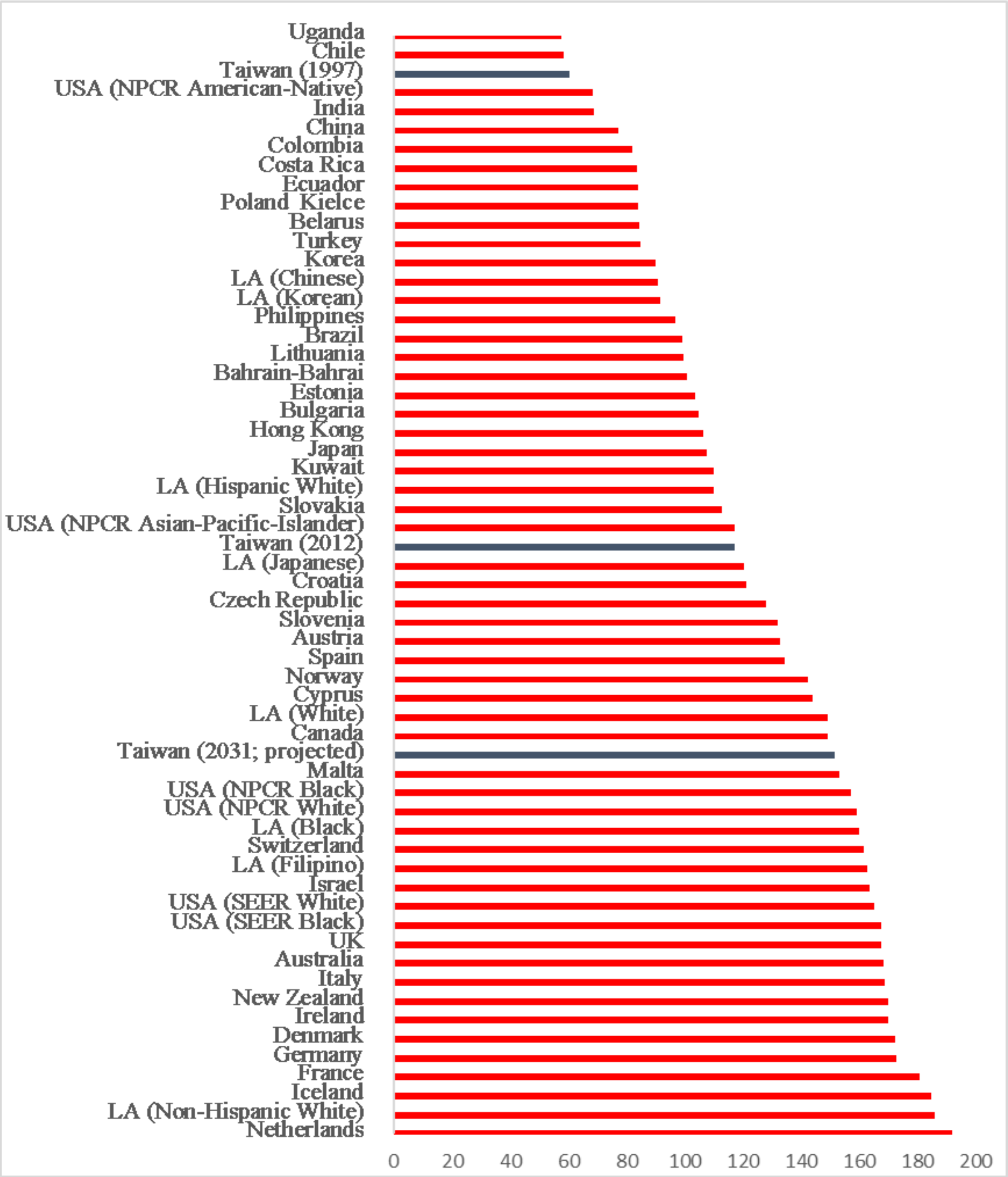

Supplementary Figure S1. Global age-standardized breast cancer incidence rates. We used the updated data from the International Agency for Research on Cancer to compare the global breast cancer age-standardized incidence rates. Most of the data are from 2012, but the available data from Slovakia and Costa Rica are from 2010 and 2011, respectively. The World Health Organization's 2000 world standard populations were used to compute the truncated age-standardized incidence rate (age range 25+ years).

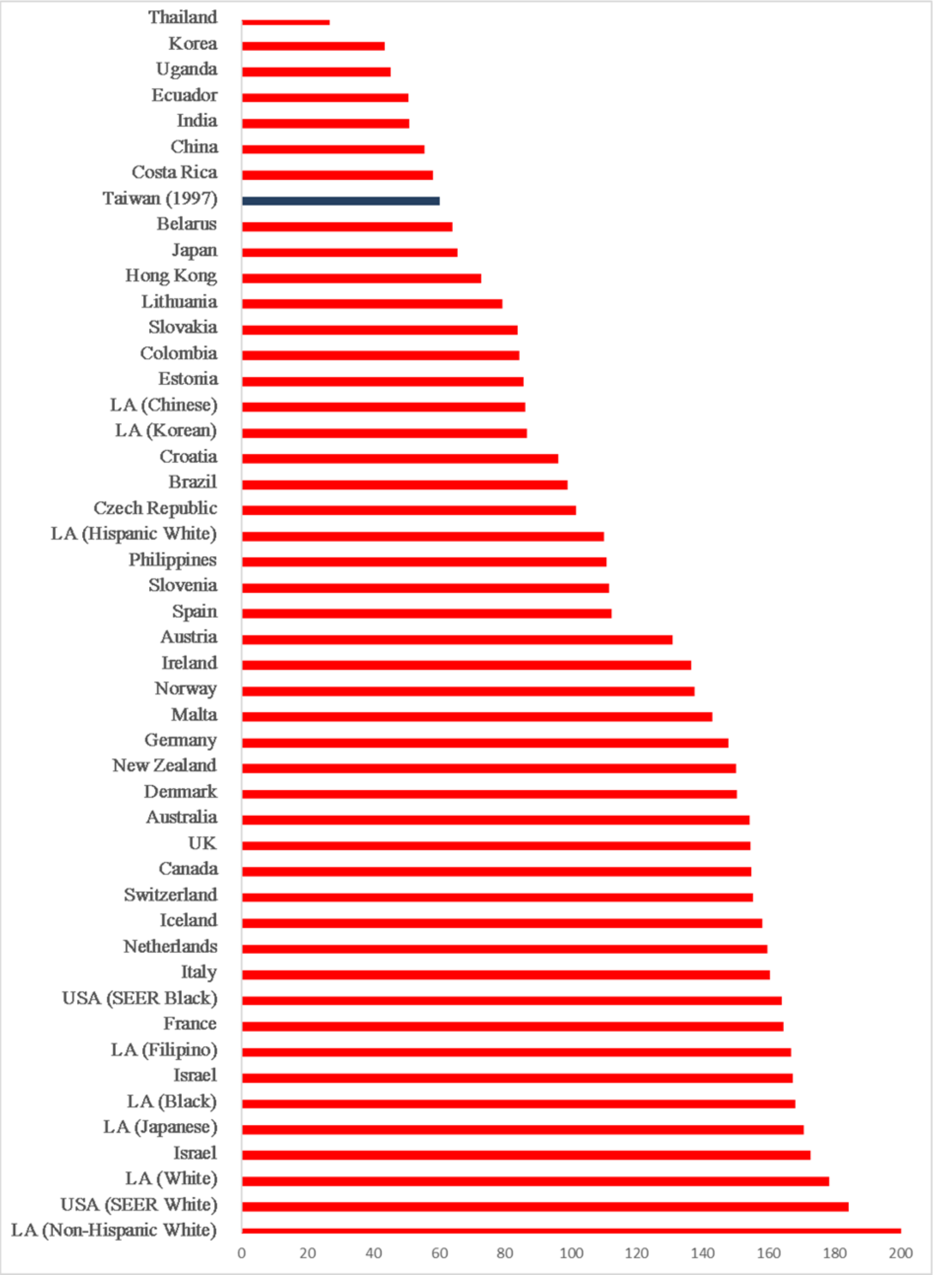

Supplementary Figure S2. Global age-standardized breast cancer incidence rates in 1997. The World Health Organization's 2000 world standard populations were used to compute the truncated age-standardized incidence rate (age range 25+ years).

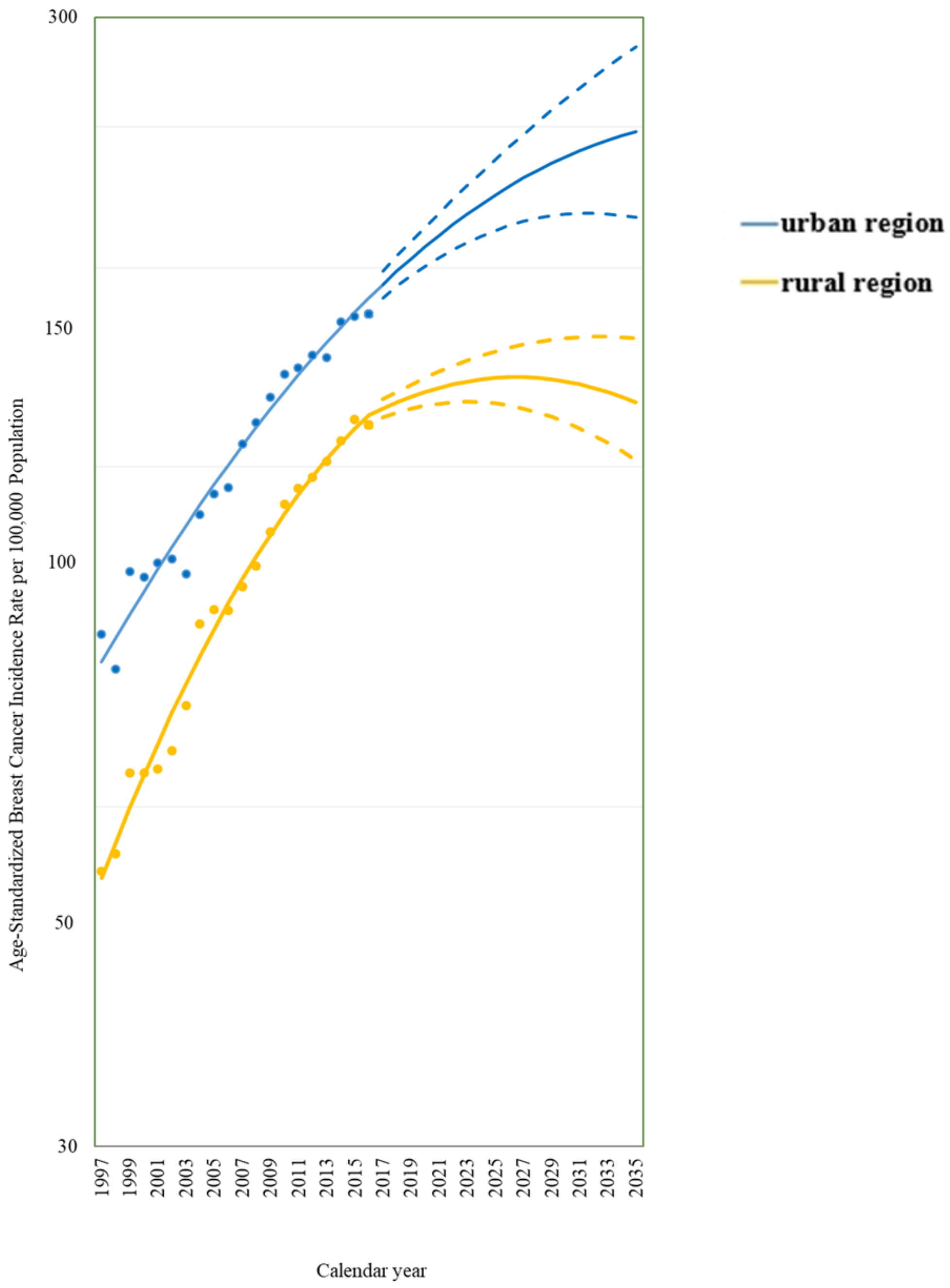

Supplementary Figure S3. Projected age-standardized breast cancer incidence rates in urban and rural regions in Taiwan.

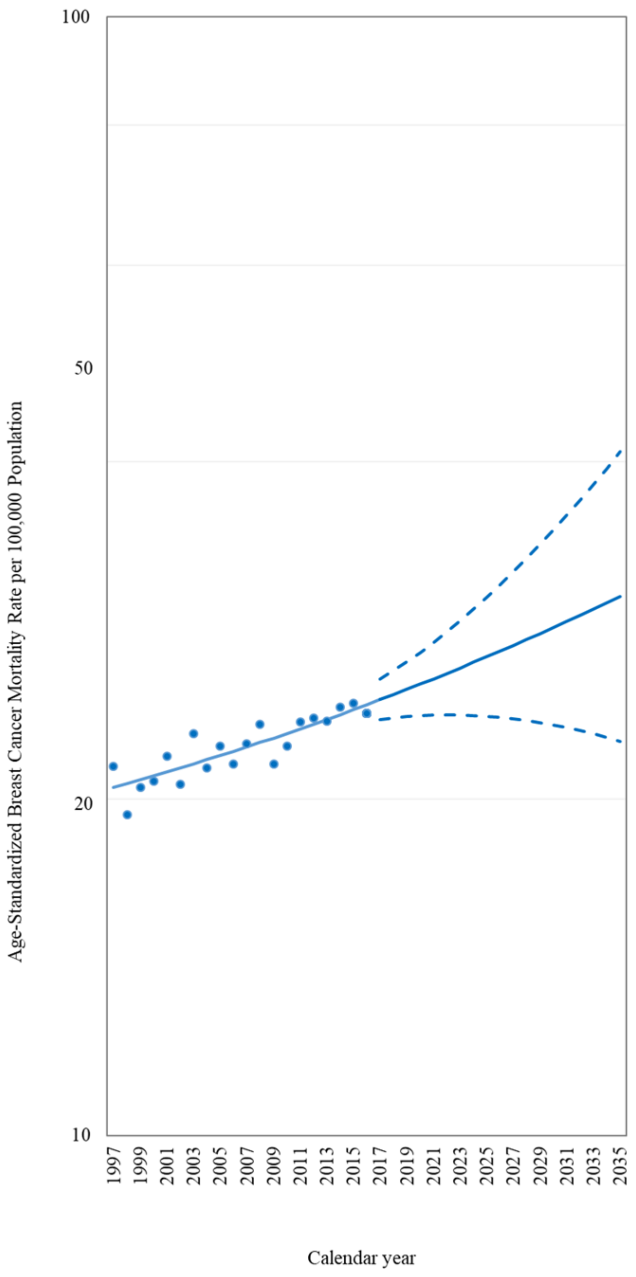

Supplementary Figure S4. Projected age-standardized breast cancer mortality rate in Taiwan.

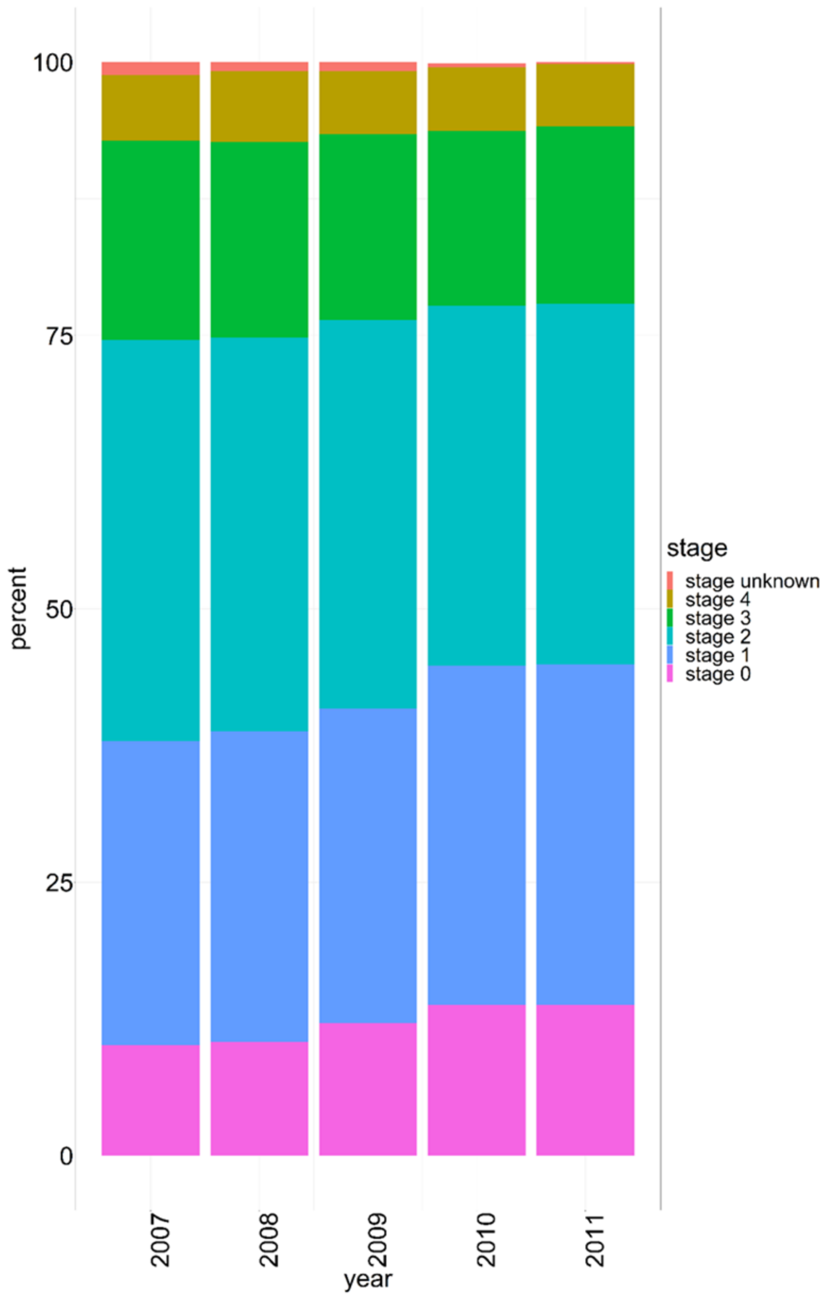

Supplementary Figure S5. Percentages of patients in various stages of breast cancer in Taiwan.

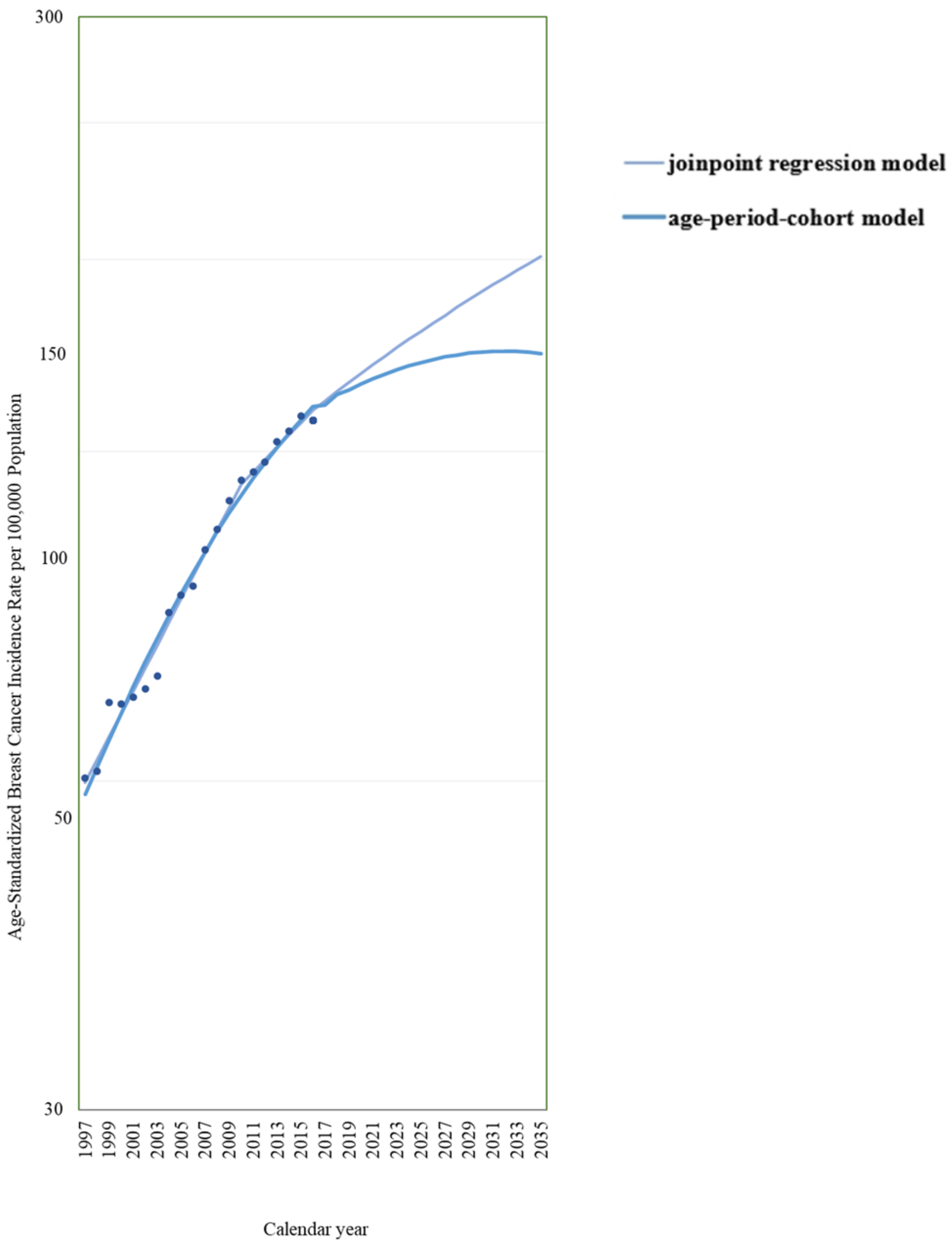

Supplementary Figure S6. Projections of breast cancer age-standardized incidence rates by the age-period-cohort model and the joinpoint regression model.
